# Visual snow syndrome: evolving neuro-optometric considerations in concussion/mild traumatic brain injury

**DOI:** 10.2217/cnc-2021-0003

**Published:** 2021-04-09

**Authors:** Kenneth J Ciuffreda, MH Esther Han, Barry Tannen, Daniella Rutner

**Affiliations:** 1 SUNY/College of Optometry, University Eye Center, Vision Rehabilitation Service, New York, NY 10036, USA

**Keywords:** concussion, diagnosis, mild traumatic brain injury, neuro-optometry, treatment, visual snow, visual snow syndrome

Visual snow syndrome (VSS) is a relatively rare, unusual and enigmatic medical condition [1–4]. It frequently occurs in patients with concussion/mild traumatic brain injury (C/mTBI) and other brain-related abnormalities [1–7]. VSS presents with a constellation of visual and non-visual problems. The hallmark symptom is the appearance of pixelated ‘visual snow’ (VS) occurring in a single plane in front of and throughout the visual field, either achromatic or chromatic in nature. Individuals diagnosed with VSS must also report two or more of the following four primary visual perceptual phenomena: photosensitivity, night vision problems (nyctalopia), palinopsia and enhanced entoptic imagery [1,2]. They frequently also report some of the following secondary visual and non-visual symptoms: photopsia, migraine, phonophobia, hyperacusis, cutaneous allodynia, tinnitus, balance disturbances and tremor [1,2].

Based on the patient’s case history and the aforementioned possible symptomatology, we have developed a VSS symptom questionnaire to assist in diagnosis, as well as to assess the effect of a therapeutic intervention [5]. This questionnaire is used in our Vision Rehabilitation Service. We have also proposed a range of basic and advanced vision tests to assist in better understanding VSS [5].

The presence of VS *per se* was reported as early as 1944 in association with the use of digitalis for heart problems [8]. However, it is only over the past decade that VSS has received considerable attention [1–7], with emphasis on C/mTBI. This has focused on defining the diagnostic criteria and related aspects. Unfortunately, there has been a paucity of reports related to treatment, which has been minimally successful; for example, the work of van Dongan *et al.* [9].

Hence we have taken a different, neuro-optometrically based, approach in our evolving clinical studies [5–7] – especially in the patient with C/mTBI – with promising results. This has included the use of specialized chromatic and achromatic tints and a saccadic tracking paradigm.

Our primary focus has involved the use of specialized spectacle tints, typically of a chromatic nature [5–7]. Different tints are tested on a patient, and the one that best reduces the perceived intensity of the VS is dispensed, being incorporated into the spectacle refractive correction. In addition, this tint typically also reduces the patient’s photosensitivity, as well as the perceived intensity of the disturbing palinopsia, if either or both are present. Two commercially available tints found to be effective are BPI-Omega (Brain Power Miami, FL, USA) and FL-41 (Brain Power Miami). In an earlier medically based study [10], the Intuitive Colorimeter™ (Cerium Visual Technologies Tenterden, Kent, UK) was used to assess chromatic tint preferences in 12 individuals with VSS. Eleven of the individuals (92%) had a distinct, repeatable chromatic preference, typically in the blue–yellow color spectrum. We have also used the Intuitive Colorimeter [7] to customize precisely the patient’s preferred chromatic filtration (‘tint’) with respect to hue and saturation, which is incorporated into their spectacle refractive correction. More recently, we have employed iPhone and computer technology, which allows for incorporation of a range of chromatic filtrations into the screen display itself [7] and thus does not require the patient to wear their chromatic spectacles for these specific tasks. However, the currently available clinical chromatic tints/filters are ‘broad band’ and therefore also reduce the overall luminance of the visual field/display screen to a moderate degree. Research by others has presented a possible solution: a thin-film, ‘notch’ filter technology [11]. This results in a very ‘narrow band’ filter that is therapeutically effective, as it targets more precisely the offensive narrow band of wavelengths, thus producing less reduction in overall luminance.

A second therapeutic approach has been directed to reduce the perceived intensity of the palinopsia, especially with trailing [6], in addition to any aforementioned positive chromatic filter effects. This involves the use of a predictable saccadic tracking paradigm [12]. We have speculated that these patients have reduced saccadic suppression [12] due to an abnormal neurological ‘disinhibition’ phenomenon [5,6]; therefore, they perceive smearing of the retinal image during a saccade. The underlying idea is to re-establish and recalibrate the system to then have normal (or near normal) saccadic suppression during execution of a saccade. Such saccadic therapy has resulted in reduction of palinopsic intensity, along with increased visual comfort, in our patients. We have further speculated that this disinhibition may reflect a more global neurological phenomenon, hence the multimodal symptomatology. The underlying neurological mechanism(s) and related anatomical substrate(s) are under investigation and remain somewhat elusive. However, a very recent study [13] suggests widespread problems in brain functional connectivity. This included dysfunctional central visual processing and broad attentional networks, which supports the aforementioned notion of a multimodal system.

## Conclusion

In conclusion, we have presented some evolving, and positive, neuro-optometric clinical approaches and considerations in patients with VSS, especially regarding the symptoms of VS, photosensitivity and palinopsia. The patient with C/mTBI and VSS now has a range of simple and effective therapeutic options, with more likely to be on the horizon as our understanding of VSS and related technology advances.
